# Vowel Identity between Note Labels Confuses Pitch Identification in Non-Absolute Pitch Possessors

**DOI:** 10.1371/journal.pone.0006327

**Published:** 2009-07-21

**Authors:** Alfredo Brancucci, Rosanna Dipinto, Ilaria Mosesso, Luca Tommasi

**Affiliations:** Department of Biomedical Sciences and Institute for Advanced Biomedical Technologies, University “G. d'Annunzio”, Chieti, Italy; James Cook University, Australia

## Abstract

The simplest and likeliest assumption concerning the cognitive bases of absolute pitch (AP) is that at its origin there is a particularly skilled function which matches the height of the perceived pitch to the verbal label of the musical tone. Since there is no difference in sound frequency resolution between AP and non-AP (NAP) musicians, the hypothesis of the present study is that the failure of NAP musicians in pitch identification relies mainly in an inability to retrieve the correct verbal label to be assigned to the perceived musical note. The primary hypothesis is that, when asked to identify tones, NAP musicians confuse the verbal labels to be attached to the stimulus on the basis of their phonetic content. Data from two AP tests are reported, in which subjects had to respond in the presence or in the absence of visually presented verbal note labels (fixed Do solmization). Results show that NAP musicians confuse more frequently notes having a similar vowel in the note label. They tend to confuse e.g. a 261 Hz tone (Do) more often with Sol than, e.g., with La. As a second goal, we wondered whether this effect is lateralized, i.e. whether one hemisphere is more responsible than the other in the confusion of notes with similar labels. This question was addressed by observing pitch identification during dichotic listening. Results showed that there is a right hemispheric disadvantage, in NAP but not AP musicians, in the retrieval of the verbal label to be assigned to the perceived pitch. The present results indicate that absolute pitch has strong verbal bases, at least from a cognitive point of view.

## Introduction

The fact that different abilities are observed as regards the identification of musical pitch has usually led to divide musicians into two categories, those who possess absolute pitch (AP), defined as the ability to name the pitch of a tone without the use of any external reference [1]–[4] and those who do not possess such an ability, commonly referred to as relative pitch musicians (RP). RP musicians typically derive the pitch of a tone by computing its interval from a reference pitch, if available. Although no cut-off has been defined between AP and RP ability, not even by psychologists of music who use dedicated tests to measure AP, it is usually reported that AP is observable in less than 20% of musicians [5] and in about 0.0001% of the total population [6]. These subjects report commonly that the identification of the correct pitch of a tone does not require any cognitive effort, and that it appears to them as a very natural and immediate skill.

The investigation of the brain processes at the basis of AP is of high interest for cognitive neuroscience as AP is a clear example of an ability which arises quite separate from other cognitive functions, thus providing a useful paradigm for understanding how specialized abilities are linked to brain processes. Until now, neuroanatomical studies have shown that the brain of AP possessors has its more prominent structural marker in the planum temporale, an area which has been regularly related to language function. These subjects have an enhanced leftward asymmetry in the size of the planum temporale, which depends on a smaller extent of the right area. In particular, the absolute size of the right planum temporale seems to be the better anatomical predictor of AP, indicating a possible pruning of the right planum temporale rather than expansion of the left in AP [7], [8]. In addition, the results of a positron emission tomography study [9] suggest the left dorsolateral prefrontal cortex as a possible additional crucial area involved in AP processing. This area plays a role in conditional associative memory, a type of memory implicated when several alternative responses to different stimuli exist and a correct response must be provided when cued by the appropriate stimulus, which is precisely the requirement of AP tasks [10]. Activation in the dorsolateral prefrontal cortex has been observed when naming tones by AP possessors but not by musicians without AP. These findings elucidate the main structural and functional bases of AP but a more precise relation between these observations and cognitive as well as perceptual functions required by the AP ability remains partially blurred.

Psychologists have investigated different aspects involved in the representation of reality and it interdependence with verbal representations. The dual-coding theory posits that humans create different mental representations starting from sensory and verbal information which are each processed along distinct channels [11]. Much evidence has shown that memory for verbal information can be strengthened by the parallel encoding of pictorial representations, but very little work has been carried out in this respect in the domain of music perception. More recent studies have elucidated new specific cognitive processes implicated in pitch identification. These include the presence of working memory and long-term representation of pitch [12]. It has been also proposed the existence of a universal internal pitch template to which subpopulations of musicians can have access through two working memory systems: a semantic associative form of memory used by AP musicians, and a more widespread form of procedural memory which allows precise access to internal pitch representations through direct vocalization [13], [14].

One of the simplest and most likely assumptions concerning cognitive bases of AP is that at its origin there would be a particularly skilled function matching the height of the perceived pitch (or even the sound frequency) to the verbal label of the presented musical tone [3], [4], [15]–[17]. This implies that the AP function is based *a)* on a frequency differential threshold (or JND, just noticeable difference) of at least one semitone, and *b)* on a capacity to associate the correct verbal label to each of the perceptual musical elements available as an effect of a). Since it has been shown that there is no difference in the JND for sound frequency between AP and NAP musicians [16], [17], the hypothesis of the present study is that the flaw in NAP musicians is purely verbal in nature and relies in a drawback in retrieving the correct label for the otherwise rightly discriminated tone height or frequency. In the Latin nomenclature, the most prominent acoustical component of the musical labels is the vowel. The hypothesis is that, when asked to identify tones, NAP musicians confuse the verbal labels to be attached to the stimulus on the basis of the vowel. Latin note names provide a suitable context to test this hypothesis as there are tone pairs sharing the same vowel (Do-Sol, Mi-Si, Fa-La, Sol-Do, Si-Mi, and La-Fa) and note pairs separated by the same musical intervals but having different vowels in their verbal labels (see Table 1). According to the hypothesis, NAP but not AP musicians should confuse more frequently e.g. a 261 Hz tone (Do) with Sol (referred to as SAME error in the present report) than e.g. a 293 Hz tone (Re) with La (referred to as DIFFERENT error in the present report) because in this last case the choice of the verbal label is not affected by the identity of the vowel which should render the association a bit more difficult. We wondered also whether this possible effect is lateralized, i.e. whether one hemisphere is more responsible than the other in the predicted confusion of notes with similar names. This question was addressed separately, by observing pitch identification during dichotic listening [18]–[23]. The hypothesis is that the right hemisphere should be responsible for such a typical verbal mismatch according to the well-known left-sided asymmetry for language processing in humans.

**Table 1:** Type of error (SAME, DIFFERENT) in the considered note pairs.

Interval	SAME	DIFFERENT	AMBIGUOUS *(not considered)*
**V**	Do-Sol, Mi-Si	Re-La, Fa-Do, Sol-Re, Sol#-Re#, La-Mi, Si-Fa#	Do#-Sol#, Re#-La#, Fa#-Do#, La#-Fa
**Major III^rd^**	Fa-La	Do-Mi, Do#-Fa, Re-Fa#, Re#-Sol, Mi-Sol#, Sol-Si, La-Do#, La#-Re	Fa#-La#, Si-Re#, Sol#-Do
**IV**	Sol-Do, Si-Mi	Do-Fa, Re-Sol, Re#-Sol#, Mi-La, Fa#-Si, La-Re	Do#-Fa#, Fa-La#, Sol#-Do#, La#-Re#
**Minor VI^th^**	La-Fa	Do#-La, Re-La#, Mi-Do, Fa-Do#, Fa#-Re, Sol-Re#, Sol#-Mi, Si-Sol	Do-Sol#, Re#-Si, La#-Fa#

Do-Sol means that a label “Sol” has been attached to a pitch of “Do” (SAME error). Ambiguous pairs refers to those pairs which can be categorized as SAME or DIFFERENT depending whether the note is perceived as sharp or flat, e.g. Do#-Sol# can be interpreted as SAME (Do#-Sol#) or DIFFERENT (Re*b*-Sol# or Do#-La*b*). These pairs were therefore not considered.

## Results

The dependent variable was the number of errors made by confusing notes with the same vowel in the label (e.g. perceiving the note Sol when a Do was presented or the note Fa when a La was presented, SAME error) and by confusing notes with different vowels in the label (e.g. perceiving the note Si when a Re was presented or the note Fa when a Do was presented, DIFFERENT error). Only pairs separated by the same musical interval were compared. For each musical interval taken into account, the number of errors was divided by the number of pairs having the same vowel or a different vowel in the label. Statistical effects were evaluated by mixed analysis of variance (ANOVA) at a significance level of p = 0.05. Since preliminary observation of the data distributions indicated that they met ANOVA criteria concerning normality and homogeneity, untransformed scores were used for the statistical analyses.

### Preliminary analyses

Preliminary statistical analyses indicated that sex and handedness of the participants did not influence statistical results in both standard and dichotic tests (no main or interaction effects). Similarly, in the dichotic test, the headphone position at the beginning of the test (upright or reversed) and the hand which was used to give the response by moving the mouse showed no significant interactions with the factor ‘ear’. These variables were therefore not included in the subsequent analyses.

### AP tests

**Figure 1:**
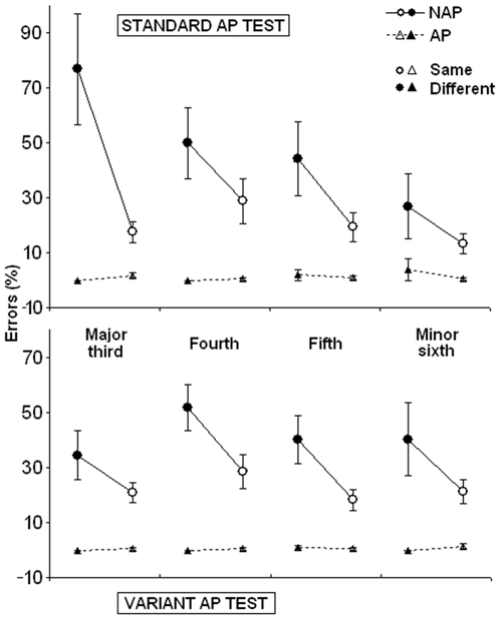
Means and standard errors in the two AP tests (standard and variant). Dependent variable is the Type of error (SAME, DIFFERENT) measure for the intervals of interest (see Table 1).

A 2×2×2×4 mixed ANOVA with Group (AP, NAP) as an between-subjects factor, Test (standard, variant), Type of error (SAME, DIFFERENT), and Interval (third, fourth, fifth, sixth) as repeated factors was carried out. Results are illustrated in Figure 1.

The ANOVA showed a significant main effect of the Group (F_1,51_ = 86.2; p<0.000001), due to a better performance (*considering only the observed intervals,* see Table 1) of the AP compared to the NAP group, a significant main effect of the Type of error (F_1,51_ = 18.5; p = 0.000077), due to a major number of SAME compared to DIFFERENT errors, and a significant interaction Group × Type of error (F_1,51_ = 18.5; p = 0.000079) due to the fact that the incidence of SAME errors was higher in the NAP group. No other main effects or interactions were observed. Tukey post-hoc analysis showed that the effect of the Type of error was significant only in the NAP group (p = 0.000165). Table 2 shows descriptive results of the Type of error variable in the two groups for both tests.

**Table 2:** Percent errors in the intervals of interest (see Tab. 1) observed in the two groups in the standard and variant tests.

	Standard test		Variant test	
	SAME	DIFFERENT	SAME	DIFFERENT
**AP**	1.1±0.8	0.9±0.3	0.2±0.2	0.7±0.3
**NAP**	48.2±7.5	18.7±3.3	42.0±6.0	21.7±3.0

### Dichotic AP test

**Figure 2:**
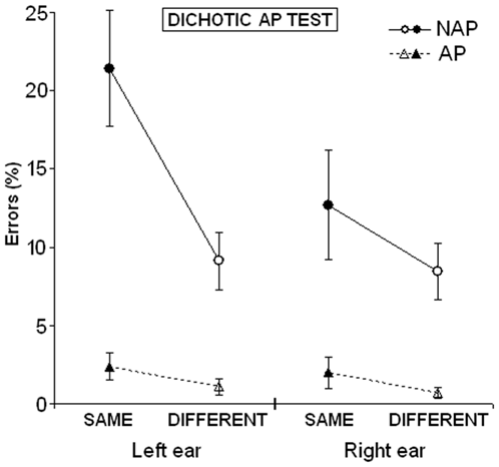
Means and standard errors in the dichotic test for the left and right ears. Dependent variable is the Type of error (SAME, DIFFERENT) considering only the intervals of interest (see Table 1).

A 2×2×2 mixed ANOVA with Group (AP, NAP) as an independent factor, and Ear (left, right) and Type of error (SAME, DIFFERENT) as repeated factors was carried out. Results are illustrated in Figure 2. Of note, in the dichotic AP test the factor Interval was not considered as the number of trials for each of the intervals of interest was too low due to the fact that the two ears had to be tested separately. Moreover, the above analysis showed that there is no effect of the interval on the type of error.

The ANOVA showed a significant main effect of the Group (F_1,43_ = 23.6; p<0.000001), due to a better performance (*considering only the observed intervals,* see Table 1) of the AP compared to the NAP group, a significant main effect of the Ear (F_1,43_ = 16.9; p<0.000188), due to the lower number of errors made with the right compared to the left ear in the intervals of interest (right ear advantage), and a significant main effect of the Type of error (F_1,43_ = 7.4; p = 0.009257), due to the higher number of SAME compared to DIFFERENT errors. First order interactions showed a significant Group × Ear effect (F_1,43_ = 9.0; p = 0.004640) due to the fact that the right ear advantage was more pronounced in the NAP group (*still considering only the observed intervals*), a significant interaction Group × Type of error (F_1,43_ = 5.3; p = 0.026551) due to the fact that the incidence of SAME errors was higher in the NAP group, and a significant Ear × Type of error interaction (F_1,43_ = 4.6; p = 0.038168) due to the fact that the incidence of SAME errors was higher for the left ear. Second-order interaction (triple interaction Group × Ear × Type of error) showed a significant effect (F_1,43_ = 4.6; p = 0.037654) indicating that the higher incidence of SAME errors for the left ear occurred mainly in the NAP group. Duncan post-hoc analysis results are reported in Table 3.

**Table 3:** Percent errors for the left and right ear in the intervals of interest (see Tab. 1) observed in the two groups in the dichotic test.

	Left ear		Right ear	
	SAME	DIFFERENT	SAME	DIFFERENT
**AP**	2.3±0.8	1.1±0.5	2.0±0.9	0.7±0.3
**NAP**	21.4±3.6	9.1±1.8	12.7±3.5	8.4±0.8

Finally, we report laterality indices, which were calculated as follows: (R−L)/(R+L)×100, were R is the number of errors of the right ear and L the number of errors of the left ear. Mean laterality indices in the NAP group were −36.6±12.5 for SAME errors and 1.7±13.9 for DIFFERENT errors. Mean laterality indices in the AP group were −7.9±11.9 for SAME errors and −3.1±11.0 for DIFFERENT errors.

## Discussion

The results of the present study indicate that the performance of NAP musicians in pitch identification tasks is strongly affected by the phonetic clues available in the note label. NAP subjects tend to confuse notes having the same vowel in their labels much more frequently than notes having different vowels in their labels, that is, they tend to make more frequently the type of error here referred to as SAME error compared to DIFFERENT error. Of note, vowels constitute the main phonetic content of the labels of musical tones, at least in terms of acoustic duration and energy amount. The result was very strong and has been obtained with two different AP-tests, one in which the verbal label of the note was visually available during the performance of the response and the other in which it was not. The fact that the two tests yielded the same results indicates that the phonetic effect of the note label does not depend upon the external (visual) information available during the selection of the response but rather that it is related to an internal, likely auditory, representation of the verbal label of the musical note. A further result showed that in NAP musicians SAME errors were more frequent when the tone was presented to the left ear, suggesting a right hemispheric disadvantage in retrieving and matching the verbal note label to a perceived musical tone height.

On the basis of the present results it can be hypothesized that during the retrieval of the note label to be attached at the perceived tone pitch, a competition occurs in NAP subjects between labels having similar phonetic content. When the choice is between two labels sharing the same vowel the level of performance is near chance level, indicating that these subjects do not posses a neural mechanism devoted to the retrieval and association of the correct phonetic elements to a perceived pitch. This view can complement and expand the two-component model proposed by Levitin and Rogers [4] according to which AP consists of a ‘pitch memory’, which is widespread in the population and a ‘pitch labelling’, which is possessed exclusively by AP subjects. According to the present results, also NAP musicians would possess a ‘pitch labelling’ mechanism, but this mechanism suffers strongly when labels containing the same vowel have to be discerned. In other words, NAP subjects can match the perceived pitch only with the vowel contained in the linguistic label.

In the present data analysis design, results from the AP group suffered possibly from a floor effect but the aim was to utilize this group only as a baseline. However the pattern of results obtained here suggests that these subjects are perhaps not affected by the phonetic content of the note labels. In addition, it seems that the stronger difference between AP and NAP musicians is in the correct retrieval of the note verbal labels depending upon their phonetic properties, at least those of the vowel. This could lead to the hypothesis that the ability to connect a tone to a label has two levels of expertise in the retrieval of the note label. One, corresponding to NAP ability, discerns just between the vowel sounds and tends to fail when the choice is between two labels having the same vocal content. The other, corresponding to AP ability, can discern between both vowel and consonant sounds, or between the whole label, and does not suffer from the identity of the vowel in the label.

The results observed here are possibly underestimated as SAME errors were always (100% of times, see Table 1) associated to mistakes in identifying musical notes corresponding to white keys of the piano whose pitch is usually easier to identify [1]. On the contrary, DIFFERENT errors were sometimes (32.1% of times, 9 pairs out of 28) associated to mistakes made in identifying musical notes corresponding to black keys, which are usually more difficult to identify. Nevertheless, NAP musicians tended to make more SAME than DIFFERENT errors.

Another point to emphasize is related to sex distribution in the two groups and to the fact that no sex differences were found in the tests. Regarding the standard test, the higher number of males in the two groups could have caused an underestimation of the main effect found in the present study, i.e. the verbal confusion between note labels. Actually, females are typically more “verbal” of males and an equal number of male and females could have emphasized the verbal effect. Regarding the dichotic test, although a lack of sex differences could seem surprising since it is known that males are more lateralized than females, dichotic listening only seldom reveal sex differences in ear asymmetry [24].

A previous report [25] on more than 2000 subjects tested online showed a pattern of results matching well the present observations. In that study, the note which was associated to a lower number of errors, in both AP and NAP musicians together, was the note D. This result, which has been observed thanks to the large sample size, can be explained along with the present evidence by reference to the fact that the solfège name of D is Re, which is the sole note that does not have a companion regarding vowel similarity. This would render the retrieval of the note label easier if compared to other notes. Of note, solfège labels are the ones which are generally associated with the corresponding tones when musicians learn solfège and singing, a process which could play a strong role in AP acquisition. Another interesting result of that study is that the note G# is the one which is more frequently misidentified, often in favour of the note A. Athos and coworkers state that “at least part of the explanation for the G# error could lie in the use of A as the universal tuning frequency. Orchestras tune to an A over a fairly wide frequency interval (…) Musicians and concert-goers are thus exposed to a wide range of tuning A pitches, and those with AP may have learned to accommodate to this broad spectrum, capturing both the presented G# and, to a lesser extent, A# (…) within the A category”. Another part of the explanation could rely in the solfège note label of G# (Sol) which suffers both from the fact that it can be confused with Do if perceived as Sol# and with Fa if perceived as La*b* and possibly also from the fact that it corresponds to a black key amid two other notes which share the vowel in the label (Fa and La).

If the present findings are not confined to Latin note names but rather describe a universal phenomenon, they would suggest that the incidence of AP in a population can be influenced by the phonetic content of the note names. Namely, note labels with several different phonetic clues would render pitch identification easier, in particular as regards the retrieval process of the label [13], increasing AP incidence. For instance, Anglo-Saxon note names have more similar labels from a phonetic point of view, providing thus fewer phonetic clues for pitch identification than Latin note names. Phonetic structure of Anglo-Saxon note labels relies mainly on the sound /i:/ (in English) which is present in the notes C, D, E, G and B, leaving only the notes F (/e/) and A (/ei/) with own phonetic clues in the vowel sound. Conversely, Latin phonetic structure of note verbal labels is more distributed, using four different phonemes (/o/, /e/, /i/, and /a/) each for two notes except than the /e/ phoneme which is present only in the Re note label. On the other hand, Latin labels have only one one-to-one relation phoneme-height, precisely /e/-Re, whereas Anglo-Saxon labels have two (F-/e/ and A-/ei/). In Asian languages such as Korean and Indian, the conventional note labels are mainly based on the vowel /a/ (Korean: Da, La, Ma, Ba, Sa, Ga, Na; Indian: Sa, Re, Ga, Ma, Pa, Dha, Ni). However, importantly, solfège notes are worldwide labelled using mostly the Latin version, thus levelling the possible effect of phonetics on AP incidence. As of now, it has been observed that AP is more frequent among Asians compared to western populations. However, the reason of this bias is unclear. The higher rate of AP among Asians is not attributable to sociocultural variables, because a comparable high AP rate is also found in Americans of Asian descent. Speaking a tonal language per se cannot account for this finding, as not all Asian languages are tonal. A possible explanation could be related to musical training. Asians are significantly more likely to have received a ‘fixed pitch training’, such as the Suzuki method, which reinforces tone/name associations method, compared with Caucasians who are trained with an interval-based learning method [26]. Further studies should investigate the possible dependence of AP incidence and ability on phonetic cues provided by note labels.

The present findings suggest an intriguing link with the fact that errors in pitch identification made by AP-musicians are mostly of one semitone [1], [3]. Actually, since the JND for tone height is smaller than one semitone [about 1/10^th^ of a semitone [27], but in musicians it is even smaller [28]], it could be speculated that in AP-musicians a purely phonetic mismatch occurs when they confuse two notes separated by one semitone. In fact, confusions between labels of notes which are one semitone apart can be often interpreted as phonetic confusions, because the label of most notes remains the same if added by a flat or a sharp. In these cases, also the phonetic clue provided by the consonant is the same (e.g. Do and Do sharp provide both the phonetic consonant cue /d/ with the vowel phoneme /o/), and this total phonetic identity could create a trouble also for AP-musicians.

Many attempts have been made in order to improve AP ability or even to teach it to people who do not possess AP at all. The results obtained in the present study could give some suggestions to those who want to “learn” AP. Since a great number of errors made by NAP musicians involves SAME errors and are caused by a right hemisphere disadvantage, it could be helpful to concentrate the training on the discrimination of notes having similar verbal labels and on the performance of the left ear, using contralateral white noise. In conclusion, the present results suggest that if each note would have a verbal label with totally different phonetic properties, then perhaps AP would be more common.

## Materials and Methods

### Participants

Fifty-two healthy subjects (36 males and 16 females) volunteered in the experiment. Mean age was 25.2 (standard deviation = 9.0). All of them were musicians recruited at the music conservatories of Bari and Pescara, Italy. They all declared to have no auditory impairment. Audiometric assessment was performed, in which subjects had to press a button when a complex tone of 264 or 395 Hz, presented via earphones repeatedly with increased intensities (steps of 2.5 dBA), became perceivable. Subjects were recruited when no (±5 dBA) different hearing thresholds were present between the left and the right ear. Mean handedness index was 59.0 (standard error = 4.6) as measured with the Italian revised version of the Edinburgh Handedness Inventory [29]. Scores were distributed as follows: 35 subjects scored ≥50, 13 subjects scored ≥0 and <50, and 2 subjects scored <0. Most of the subjects claimed to possess AP when informally interviewed. Subjects were assigned to two groups (absolute pitch group, AP, and non-absolute pitch group, NAP) on the basis of their average performance in the tests. Half of the subjects (26 individuals) scoring the lowest distance from target (in semitones) were assigned to the AP group, the other half (26 individuals) were assigned to the NAP group. Mean±s.e.m. performance in the AP group was 0.08±1.89 semitones deviation from target, whereas mean performance in the NAP group was 0.58±10.83 semitones deviation from target. The AP group was composed of 19 males and 7 females, the NAP group was composed of 17 males and 9 females. Mean handedness was 60.2±4.8 in the AP group and 57.8±4.3 in the NAP group. Finally, both musical expertise and musical education starting age were not statistically different between the two groups. The mean number of years of music education was 17.0±2.1 years in the AP group and 17.4±1.64 years in the NAP group (p = 0.87). The mean musical education starting age was 8.2±0.7.1 years in the AP group and 7.8±0.8 years in the NAP group (p = 0.74). Subjects gave their verbal informed consent to participate in the experiments. The study was approved by the committee of the University of Chieti and Pescara which deemed it sufficient to obtain verbal consent rather than written consent.

### AP-tests

**Figure 3:**
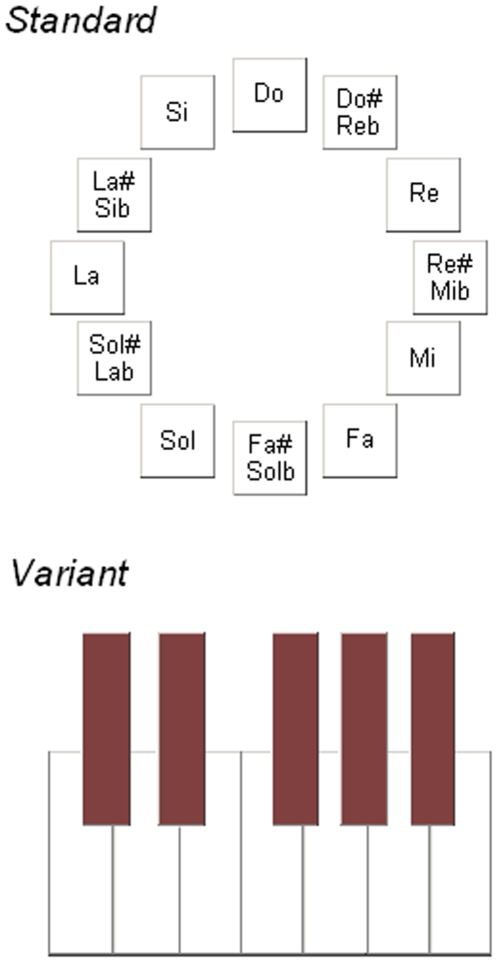
Subjects gave their response by clicking with the mouse on the corresponding note presented as showed here in the standard AP test (top) and in the variant AP test (bottom).

Subjects were presented with a standard AP test, which was borrowed with permission from Robert J. Zatorre at BRAMS (www.brams.org) and with a variant of it. The tests consisted of 108 trials in which subjects had to identify the name of the presented musical tone, which changed at every trial. Tone height ranged from a3 to a5, tone duration was 1 s, and intensity could be 67, 70, or 73 dBA. Spectral composition was harmonic, with eight spectral components having the following relative amplitudes: 1, 0.7, 0.5, 0.2, 0.15, 0.05, 0.02, 0.01. Sampling rate of each sound was 44100 kHz and amplitude resolution 16 bit. Amplitude envelope of the tones contained 50 ms rise and fall times. The response was given by mouse click on the (perceived) note label selected among the 12 note labels arranged in a circle on the computer screen. The note names Do, Do#/Re*b*, Re, Re#/Mi*b*, Mi, Fa, Fa#/Sol*b*, Sol, Sol#/La*b*, La, La#/Si*b*, and Si were arranged in a circle in which the note name “Do” was at the top, Fa#/Sol*b* at the bottom, Re#/Mi*b* on the right and La on the left. The only difference in the variant test was that the computer screen displayed one octave of a keyboard (12 keys, without labels) instead of the note names arranged in a circle, and the subject had to click on the key corresponding to the perceived musical note (Figure 3). The mouse arrow was automatically positioned at the centre of the circle at the beginning of each trial in the standard test and in the upper left corner or in the lower right corner (in alternation) of the keyboard in the variant test. The variant test was designed in order to control for a possible verbal contamination in the selection of the response due to the visually presented note labels.

### Dichotic AP-test

Forty-four subjects out of the 52 who took part in the standard and variant AP-test volunteered also in the dichotic test. In this test, tones corresponding to all notes comprised in the range from C3 to B5 were presented. The duration of the tones was 400 ms and the intensity level 70 dBA. Spectral composition was harmonic, with eight spectral components having the following relative amplitudes: 1, 0.7, 0.5, 0.2, 0.15, 0.05, 0.02, 0.01. One dichotic pair consisted in one of the musical tones and contralateral white noise. The white noise was presented at 75 dBA and had the same duration of the contralateral tone. Sampling rate of each sound was 44100 kHz and amplitude resolution 16 bit. Amplitude envelope of both tones and white noise contained 50 ms rise and fall times. To obtain a dichotic pair, a tone and white noise were aligned on the two auditory channels by means of the CSound programming language [30]. The test consisted in a sequence of 120 dichotic pairs composed of a musical tone presented at one ear and white noise presented at the other ear. In each trial, after the presentation of the dichotic pair, the task of the subject was to indicate with the mouse the note perceived at the ear receiving the musical tone. The display showed the same picture as the standard test described above. The 120 trials were grouped into 20 blocks of 6 trials each. The blocks were separated by a 4-s interval and each block was preceded by a beep (2000 Hz, 200 ms) presented monaurally to the ear that was about to receive the target stimuli in that block. Subjects were instructed to direct their attention to the side of the monaural beep in the subsequent block (dichotic test with focused attention) and were informed that tones would be delivered to that side. In half of the blocks tones were presented to the right ear and white noise was presented to the left ear, in the other half vice versa. Tones were allocated to blocks on a pseudorandom basis. The side (ear) of presentation of the target stimulus changed at every block. Subjects were familiarized with the test by listening to a sample sequence consisting of 10 dichotic pairs. The format of the test was chosen because it allowed us to control the direction of attention, i.e. fluctuations of attention from one to the other ear are minimized [31]–[34]. The experiment was completely automated by means of an ad hoc software written in Microsoft Visual Basic. Stimuli were processed by means of a PC with Sound Blaster audio card (Creative, Model AWE 32). Subjects wore headphones (Sennheiser HD 202) and sat comfortably in front of a computer monitor with one hand laying on the computer mouse, which was used to indicate on the screen the name of the note which was perceived in each dichotic pair. Half of the subjects used the right hand and half used the left hand to move the mouse. Subjects were instructed to look at the note array in the centre of the screen in front of them and to avoid shifting their gaze laterally during the experiment.

The intensity level of the sounds was the same at both earphones, as measured by a phonometer. However, in order to keep maximal control on the intensity level of the stimuli presented at the ears, after 60 trials (middle of test) subjects reversed the two earphones (the initial position of the headphone was counterbalanced across subjects). Selected note names and latency of response were automatically stored for later analysis.

### Data analysis

The aim of the present study was to analyze whether when subjects make an error in identifying a musical tone they tend to confuse the corresponding note label more often with another note label containing the same vowel (SAME error) than with a tone label containing a different vowel (DIFFERENT error). The note pairs that can be confused on the basis of the vowel are Do-Sol and Mi-Si (ascending fifth or descending fourth interval error), Fa-La (ascending major third or descending minor sixth error), Sol-Do and Si-Mi (ascending fourth or descending fifth error) and La-Fa (ascending minor sixth or descending major third error). The number of errors made by confusing each of the above note pairs (SAME error) was compared with the number of errors made by confusing all possible note pairs having the same musical interval (DIFFERENT error). For instance, the number of errors made by responding Sol when Do was presented and by responding Si when Mi was presented (SAME error, fifth interval) was compared with the number of confusions made by responding La when Re was presented, Re when Sol was presented, and Mi when La was presented (DIFFERENT error, fifth interval). Similar comparisons were made for the other SAME pairs Fa-La, Sol-Do, and Si-Mi which were compared with DIFFERENT note pairs separated by identical intervals (see Table 1). It is worth mentioning that pairs containing a note which is usually labelled in two different ways (i.e. Re#/Mi*b*) were not considered if one of the two possible labels changed the pair from SAME to DIFFERENT or vice versa. That is, for instance, the pair Re#/La# (a possible control of Do/Sol, fifth interval) was not considered as it can be labelled as DIFFERENT (Re#/La#, Re#/Si*b*, Mi*b*/La#) but also as SAME (Mi*b*/Si*b*).

A similar data analysis was carried out for the dichotic AP test, separately for each ear. The number of SAME errors was then divided by the number of SAME pairs (having same vowel in the verbal label) and the number of DIFFERENT errors was divided by the number of DIFFERENT pairs (having different vowel in the verbal label) for each interval of interest, i.e. major third, forth, fifth, and minor sixth.
